# Verification of the predictive value of EV-associated biomarkers MMP9 and CEACAM1 in rehabilitation of ischemic stroke

**DOI:** 10.20517/evcna.2025.122

**Published:** 2026-04-13

**Authors:** Jiao Luo, You Cai, Yanling Cai, Chunxia Zhang, Ankang Liu, Yongyang Huo, Xuehui Fan, Ruixue Ye, Hong Gao, Meiling Huang, Xiaohua Zhang, Mingchao Zhou, Yulong Wang

**Affiliations:** ^1^Department of Rehabilitation Medicine, Dapeng New District Nan’ao People’s Hospital, the First Affiliated Hospital of Shenzhen University, Shenzhen 518100, Guangdong, China.; ^2^Department of Rehabilitation Medicine, the First Affiliated Hospital of Shenzhen University/the Second People’s Hospital of Shenzhen, Shenzhen 518035, Guangdong, China.; ^3^Department of Cell Biology and Neuroscience, Division of Life Sciences, School of Arts and Sciences, Rutgers, The State University of New Jersey, Piscataway, NJ 08854, USA.; ^4^Shenzhen Institute of Translational Medicine, the First Affiliated Hospital of Shenzhen University/the Second People’s Hospital of Shenzhen, Shenzhen 518038, Guangdong, China.; ^5^Shenzhen Institute of Translational Medicine, the First Affiliated Hospital of Shenzhen University, Shenzhen Second People’s Hospital, Shenzhen SecreTech Co., Ltd., Shenzhen 518129, Guangdong, China.; ^6^Department of Pharmacy, the Eighth Affiliated Hospital of Sun Yat-sen University, Shenzhen 518000, Guangdong, China.; ^#^These authors contributed equally to this work.

**Keywords:** Ischemic stroke rehabilitation, MMP9, CEACAM1, extracellular vesicles, biomarkers

## Abstract

**Aim:** Extracellular vesicles (EVs) contribute to stroke rehabilitation by mediating intercellular signaling during inflammation and tissue repair. Here we report EV-associated surface proteins as potential biomarkers for predicting recovery of activities of daily living (ADL) during the subacute phase of ischemic stroke (IS).

**Methods:** IS patients and healthy controls (HCs) were recruited for this study, with serum samples analyzed across three study stages. In the discovery subset (10 IS, 6 HCs), serum proteomics was used to identify differentially expressed proteins (DEPros) and perform Gene Ontology (GO) enrichment analysis. In the exploration subset (7 IS, 12 HCs), a proximity-dependent barcoding assay (PBA) was employed to profile surface proteins on individual EVs and screen for biomarker candidates. In a validation cohort, patients were grouped by ADL improvement (little-effect recovery, LE, *n* = 30; obvious-effect recovery group, OE, *n* = 22) based on Longshi Scale and Barthel Index assessments at admission and at 3 months follow-up. Targeted biomarker validation was performed with enzyme-linked immunosorbent assay (ELISA) and receiver operating characteristic (ROC) analysis.

**Results:** A total of 113 DEPros were identified, with GO term enrichment in EV-related pathways. PBA profiling revealed matrix metalloproteinase 9 (MMP9), carcinoembryonic antigen-related cell adhesion molecule 1 (CEACAM1), melanoma cell adhesion molecule (MCAM), and gelsolin (GSN) as candidate biomarkers. In the validation cohort, MMP9 and CEACAM1 were significantly elevated in the LE group. ROC analysis showed area under the curve (AUC) of 0.726 for MMP9 and 0.700 for CEACAM1 in distinguishing LE from OE.

**Conclusion:** Elevated serum levels of EV-associated biomarkers MMP9 and CEACAM1 were associated with poor ADL recovery, supporting their potential as prognostic biomarkers for stroke rehabilitation outcomes.

## INTRODUCTION

Infarct lesion volume, stroke subtype, and the National Institutes of Health Stroke Scale (NIHSS) have been demonstrated to be established predictive indices for acute ischemic stroke (IS) severity^[1-3]^. However, their prognostic value declines significantly in the subacute or convalescent phase due to the dynamic progression of stroke and patient heterogeneity. In particular, the recovery of activities of daily living (ADL) during the subacute phase is modulated by ongoing inflammation, tissue remodeling, and neuroplastic changes. Therefore, there is an urgent need to explore biomarkers that can capture these evolving pathophysiological processes and predict functional outcomes.

Extracellular vesicles (EVs), nanoscale lipid bilayer-bound particles (30-1,000 nm), have emerged as promising candidate biomarkers. EVs are widely present in peripheral blood and encapsulate diverse bioactive cargo, such as proteins, nucleic acids, and other functional biomolecules. They play pivotal roles in intercellular communication and disease progression. Accumulating evidence suggests that EVs may serve as diagnostic and prognostic biomarkers in various diseases, including Alzheimer’s disease^[4]^, stroke^[5]^, atherosclerosis^[6]^, obesity^[7]^, tumors^[8]^, and others. In the context of stroke, EVs originating from the injured brain can cross the blood-brain barrier (BBB) and enter the cerebral blood flow, offering a non-invasive window into central nervous system pathology. However, the intrinsic heterogeneity of EVs presents a major challenge, as only a small subset of vesicles may be functionally or diagnostically relevant. Therefore, sensitive and specific strategies to characterize disease-related EV subpopulations are urgently needed.

Recent advances in proximity-dependent barcoding assay (PBA) can addressed this limitation by enabling high-throughput, multiplexed profiling of surface proteins on individual EVs using microliter-scale biological samples^[9,10]^. This technique utilizes antibody-DNA conjugates and next-generation sequencing to resolve protein expression patterns at the single-EV level. PBA has been successfully applied in the identification of novel EV surface protein markers in several conditions, such as Alzheimer’s disease, aging, and colorectal cancer^[10]^. Applying this cutting-edge technology to stroke rehabilitation may yield new insights into the molecular basis of recovery and facilitate the discovery of EV-based prognostic indicators.

In the present study, we aimed to identify EV-associated surface proteins that are predictive of rehabilitation outcomes in patients with IS. By integrating high-resolution proteomic techniques with liquid chromatography tandem-mass spectroscopy (LC-MS/MS) and single-EV profiling using PBA, we analyzed serum-derived EVs from IS patients and healthy controls (HCs). We believe that these EV-associated surface proteins hold significant potential as prognostic biomarkers for ADL recovery and may inform the development of targeted strategies to enhance stroke rehabilitation.

## METHODS

### Study participants

This study was a prospective observational cohort conducted in the Department of Rehabilitation Medicine (72 beds) of a tertiary comprehensive hospital between June 2020 and June 2022. A total of 52 male patients in the subacute phase of IS and 20 age-matched male HCs were enrolled. The study protocol was approved by the Ethics Committee of the First Affiliated Hospital of Shenzhen University (Approval No. 20211025002-FS01). Written informed consent was obtained from all participants, and the study adhered to the principles of the Declaration of Helsinki (2013 revision). Male patients were included in this study if they met the following criteria: diagnosed with IS at admission by computed tomography (CT) or magnetic resonance imaging (MRI); aged 40-85 years; and categorized as bedridden according to ADL assessment using the Longshi Scale. Patients were excluded if they had severe systemic or mental disorders; other causes of brain injury, including blunt force trauma or penetrating head injury; or were lost to follow-up for ADL assessment. Medical history and demographic data were obtained at enrollment. HC participants were recruited concurrently from male individuals undergoing routine physical examinations in the hospital’s Health Examination Department. To ensure comparable baseline characteristics, HCs were age-matched to IS patients and rigorously screened to exclude any history of stroke, transient ischemic attack, or other major neurological, inflammatory, or malignant conditions. Individuals with pre-existing neurological deficits, active autoimmune or infectious diseases, malignancy, or inability to provide consent were excluded.

The effect of rehabilitation on ADL in the enrolled patients with IS was evaluated after 3 months of conventional rehabilitation using the Longshi Scale and Barthel Index (BI). As a supplementary measure to the modified Rankin Scale and BI, the Longshi Scale stratifies individuals with disability into the Bedridden, Domestic, and Community categories according to daily activity ability and activity range^[11]^. Patients were assigned to the little-effect recovery group (LE) if they remained in the Bedridden category from admission to the 3-month assessment, whereas those initially classified as Bedridden but reassigned to the Domestic or Community category after 3 months were included in the obvious-effect recovery group (OE).

**Figure 1:**
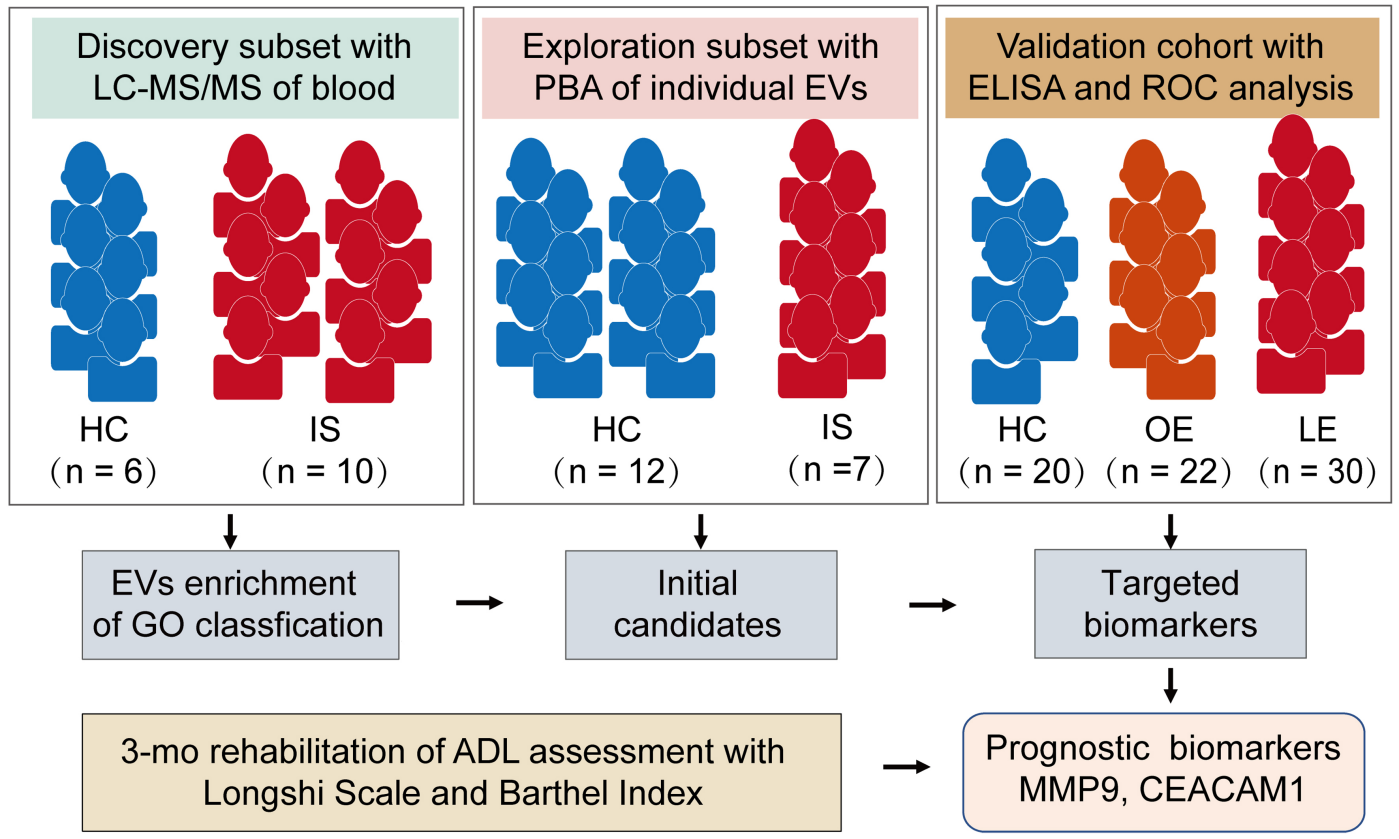
Overall experimental design for biomarkers of stroke prognosis. This is a single-center, observational, multi-stage study conducted between June 2020 and June 2022. Patients with IS were categorized into LE and OE prognosis groups according to ADL assessments using the Longshi Scale and BI at admission and after 3 months of rehabilitation. The discovery subset underwent LC-MS/MS proteomic analysis to clarify stroke-associated pathway enrichments. Surface protein profiling of individual EVs using PBA was conducted in the exploration subset to screen initial candidates. Finally, ELISA and ROC analyses in an expanded cohort were performed to validate the sensitivity and specificity of the targeted biomarkers in distinguishing between the LE and OE groups. LC-MS/MS: Liquid chromatography-tandem mass spectrometry; PBA: proximity barcoding assay; ELISA: enzyme-linked immunosorbent assay; ROC: receiver operating characteristic; HCs: healthy controls; IS: ischemic stroke; OE: obvious-effect recovery group; LE: little-effect recovery group; ADL: activities of daily living; MMP9: matrix metalloproteinase 9; CEACAM1: carcinoembryonic antigen-related cell adhesion molecule 1.

Among the 52 IS patients, 30 were categorized as LE (poor prognosis) and 22 as OE (favorable prognosis). The overall study design comprised three stages: (i) a discovery subset (LC-MS/MS serum proteomics; 10 IS *vs*. 6 HCs); (ii) an exploration subset (single-EV surface proteome profiling via PBA; 7 IS *vs*. 12 HCs); and (iii) a prospective validation cohort evaluating 3-month functional outcomes (Longshi Scale and BI) and biomarker quantification by enzyme-linked immunosorbent assay (ELISA) (LE, *n* = 30; OE, *n* = 22; HCs, *n* = 20), as illustrated in Figure 1.

### Collection of serum samples

Blood samples of the patients were obtained on the second day after hospitalization. Samples were promptly processed following the standardized protocol recommended by the Human Proteome Organization (HUPO) Human Proteome Project. Briefly, blood was drawn into plastic tubes without anticoagulant and placed at room temperature for 1 h. Subsequently, the blood samples were centrifuged at 4 °C and 3,000 × *g* for 10 min; the aliquots were stored at -80 °C until further proteomic analysis, PBA, or ELISA.

### LC-MS/MS and proteomic analysis

Proteomic profiling of serum samples was performed using LC-MS/MS. Serum samples from the discovery cohort, including 10 patients with IS (comprising 5 LE and 5 OE) and 6 HCs derived from our previously established datasets^[12]^, were analyzed. Sample preparation, LC-MS/MS acquisition, and primary data processing were conducted at Jingjie PTM BioLab (Hangzhou, China).

High-abundance serum proteins were depleted using the Pierce^TM^ Top 14 Abundant Protein Depletion Spin Columns Kit (Thermo Fisher Scientific). The remaining proteins were reduced, alkylated, and digested with sequencing-grade trypsin. Peptides were dissolved in solvent A (0.1% formic acid in water) and separated on an EASY-nLC 1200 UPLC system (Thermo Fisher Scientific) using a reverse-phase analytical column. Peptides were eluted with a linear gradient of solvent B (0.1% formic acid in acetonitrile) as follows: 4%-20% over 68 min, 20%-32% over 14 min, 32%-80% over 4 min, followed by holding at 80% for 4 min, at a constant flow rate of 500 nL/min.

Mass spectrometric analysis was performed on an Orbitrap Exploris 480 mass spectrometer (Thermo Fisher Scientific) equipped with a nano-electrospray ion source operated at 2.3 kV. Field Asymmetric Waveform Ion Mobility Spectrometry (FAIMS) was applied with compensation voltages of -45 and -70 V. Full MS scans were acquired in the Orbitrap over an m/z range of 400-1,200 at a resolution of 60,000. Data-dependent MS/MS acquisition was performed at a resolution of 30,000 with a fixed first mass of 110 m/z. The top 15 most intense precursor ions were selected for fragmentation using higher-energy collisional dissociation with a normalized collision energy of 27%. Dynamic exclusion was set to 30 s. The automatic gain control (AGC) target was set to 75%, with an intensity threshold of 10,000 ions/s and a maximum injection time of 100 ms.

Raw MS/MS data were processed using Proteome Discoverer software (version 2.4.1.15). Spectra were searched against the Homo sapiens UniProt reference proteome database (release 20201214, 75,777 entries) concatenated with a reverse decoy database. Trypsin was specified as the cleavage enzyme, allowing up to two missed cleavages. The false discovery rate (FDR) for peptide-spectrum matches and proteins was controlled at < 1%. Protein quantification was performed using a label-free relative quantification approach. Abundance values were normalized by median normalization across samples and log-transformed prior to downstream analysis. The mass spectrometry proteomics data have been deposited to the ProteomeXchange Consortium via the PRIDE repository with the dataset identifier PXD052579.

Differentially expressed proteins (DEPros) between groups were identified using a threshold of *P* < 0.05 and |log2 fold change| ≥ 1. Hierarchical clustering analysis was conducted using the R package pheatmap (v1.0.12), and visualization was performed with ggplot2 (v3.5.1). Gene Ontology (GO) enrichment analysis of DEPros was carried out using the clusterProfiler R package (v4.12.6) with Benjamini-Hochberg correction (adjusted *P* < 0.05) and a simplify cutoff of 0.5. All other parameters were set to default unless otherwise specified.

### Characterization of EVs from human serum

EVs were isolated from human serum using a standard ultracentrifugation protocol. Briefly, serum samples were collected from IS patients and healthy individuals, then centrifuged at 10,000 × *g* for 10 min to remove cell debris and large particles. The serum was subsequently ultracentrifuged at 100,000 × *g* for 2 h at 4 °C, and the resulting EV pellet was resuspended in PBS. EV morphology was examined by transmission electron microscopy (TEM) (Hitachi, HT-7700). Particle size distribution was analyzed by nanoparticle tracking analysis (NTA) using a ZetaView instrument (Particle Metrix, ZetaVIEW S/N 17-310). For NTA measurements, EVs isolated from 100 μL of serum from IS patients were diluted 200-fold prior to analysis. EV identity and sample purity were further evaluated by western blotting for EV-enriched markers (CD63 and TSG101), a negative marker for cellular contamination (CALNEXIN).

### Single-EV surface protein profiling using PBA

The serum samples from the exploration group were transported with dry ice to SecreTech Co., Ltd. for surface protein profiling of single EV using PBA^[9]^. For surface protein profiling, a panel of oligonucleotide-tagged antibodies targeting EV-associated proteins was constructed by SecreTech Co., Ltd (PBA probes, Supplementary Table 1). The antibodies directed against surface proteins on EVs under investigation included typical EV biomarkers (CD63, CD9, and CD81) and a panel of cell adhesion molecules, which are broadly expressed EV membrane proteins that mediate EV targeting, cellular internalization, and signaling. Each antibody was chemically conjugated to a unique DNA oligonucleotide containing a protein-specific barcode (proteinTag) and a molecule-specific random sequence (moleculeTag), allowing protein identity and molecular abundance to be encoded simultaneously.

To enable unique labeling of individual EVs, rolling circle amplification (RCA) was employed to generate DNA nanoclusters (“complexTags”) bearing multiple tandem repeats of unique sequences. These RCA products served as molecular barcodes for distinct EVs. Following probe incubation with EVs for 1 h at room temperature, the samples were washed three times with 150 μL of PBS containing 0.05% Tween 20. Then, 2 μL of the complexes was captured in each microwell of a cholera toxin subunit B (CTB)-coated 96-well plate to achieve sparse and spatially separated EV immobilization. Subsequent hybridization of antibody-conjugated oligonucleotides to RCA products brought the proteinTags in proximity to complexTags, enabling polymerase-mediated extension reactions that physically link EV identity (complexTag) with surface protein identity (proteinTag). Polymerase chain reaction (PCR) amplification was then conducted to enrich the tagged constructs, which were subjected to next-generation sequencing.

Sequencing reads were computationally demultiplexed and matched to their corresponding proteinTag and complexTag combinations. This enabled quantitative decoding of the surface protein composition of individual EVs. Quality control metrics, including the number of moleculeTags per EV and the frequency of multiplex detection events, were employed to ensure the specificity of the profiling and to filter background signals. The PBA tests were designed according to the protocols published by Vesicode AB (Solna, Sweden) and were performed by Secretech (Shenzhen, China).

### Data processing of PBA and analysis

All analyses were performed in R (v4.4.2) using RStudio. Because the raw EV proteomics data were represented as highly sparse expression matrices and were susceptible to background interference, we applied a stringent quality-filtering step prior to downstream analysis. Specifically, EVs were retained only if they contained at least two proteins with detected expression ≥ 2 (Filter 2.2). Filtered matrices were then analyzed using the Seurat package (v4.4.0). Data normalization and variance stabilization were conducted with the SCTransform algorithm. For comparisons across samples, batch effects were corrected and datasets were integrated using Harmony, followed by dimensionality reduction and visualization with uniform manifold approximation and projection (UMAP)^[13-16]^. For each identified subcluster, differential expression markers were computed using the Wilcoxon rank-sum test implemented in Seurat’s FindMarkers function. Marker proteins were considered significant if they met both criteria: an average log2 fold change (avg log2FC) ≥ 0.25 and a Benjamini-Hochberg-adjusted *P* value < 0.05 in comparisons between the IS and HC groups.

### Validation of targeted biomarkers

Serum concentrations of carcinoembryonic antigen-related cell adhesion molecule 1 (CEACAM1), matrix metalloproteinase 9 (MMP9), and melanoma cell adhesion molecule (MCAM) were measured using commercially available human ELISA kits (Reddot Biotech, Canada; RDR-CEACAM1-Hu, RDR-MMP9-Hu, and RDR-MCAM-Hu) following the manufacturer’s instructions. Briefly, serum samples were diluted 1:10 with sample diluent. Standards were prepared by serial dilution of the reconstituted stock solution. One hundred microliters of standards and diluted samples were added to antibody-precoated 96-well plates and incubated at 37 °C for 90 min. After incubation with Detection Solution A and Detection Solution B (45 min each at 37 °C), the wells were washed and developed with TMB substrate for 15-25 min in the dark. The reaction was terminated by adding stop solution, and absorbance was read at 450 nm using a microplate reader. All samples were assayed in duplicate, and the mean optical density values were used to construct standard curves and calculate concentrations.

Patients were categorized into two groups (OE and LE group) according to their 3-month functional outcome on the Longshi Scale. Receiver operating characteristic (ROC) analyses were therefore based on this binary classification (OE *vs*. LE), with serum protein levels used as continuous predictors. ROC analysis and visualization were performed using the pROC R package (Display and Analyze ROC Curves) (version 1.18.5) and multipleROC R package (version 0.1.1), and the area under the curve (AUC) was calculated to evaluate the discriminatory ability of each biomarker.

### Statistical analysis

Clinical data were analyzed with GraphPad Prism (Version 8.0.1). Normally distributed continuous variables are expressed as mean ± SD. Data were compared by a two-tailed unpaired Student’s *t*-test or one-way analysis of variance (ANOVA) for multiple comparisons. Non-normally distributed data are expressed as the median (interquartile range, IQR), and two-group comparisons were performed using the Wilcoxon rank-sum test. Correlations between candidate biomarker levels and age were assessed using Pearson correlation analysis. To further evaluate the independent associations of EV-associated biomarkers with functional outcome, a multivariable binary logistic regression model was performedwith Age, BI_admission, MMP9, and CEACAM1 entered as covariates, and odds ratio (ORs) with 95% confidence intervals (CIs) were reported. Differences were considered statistically significant at *P* value < 0.05; ^*^represents *P* < 0.05, ^**^represents *P* < 0.01, ^***^P < 0.001, and ^****^represents *P* < 0.0001.

## RESULTS

### Robust pathologic alterations in EVs associated with IS rehabilitation

**Figure 2:**
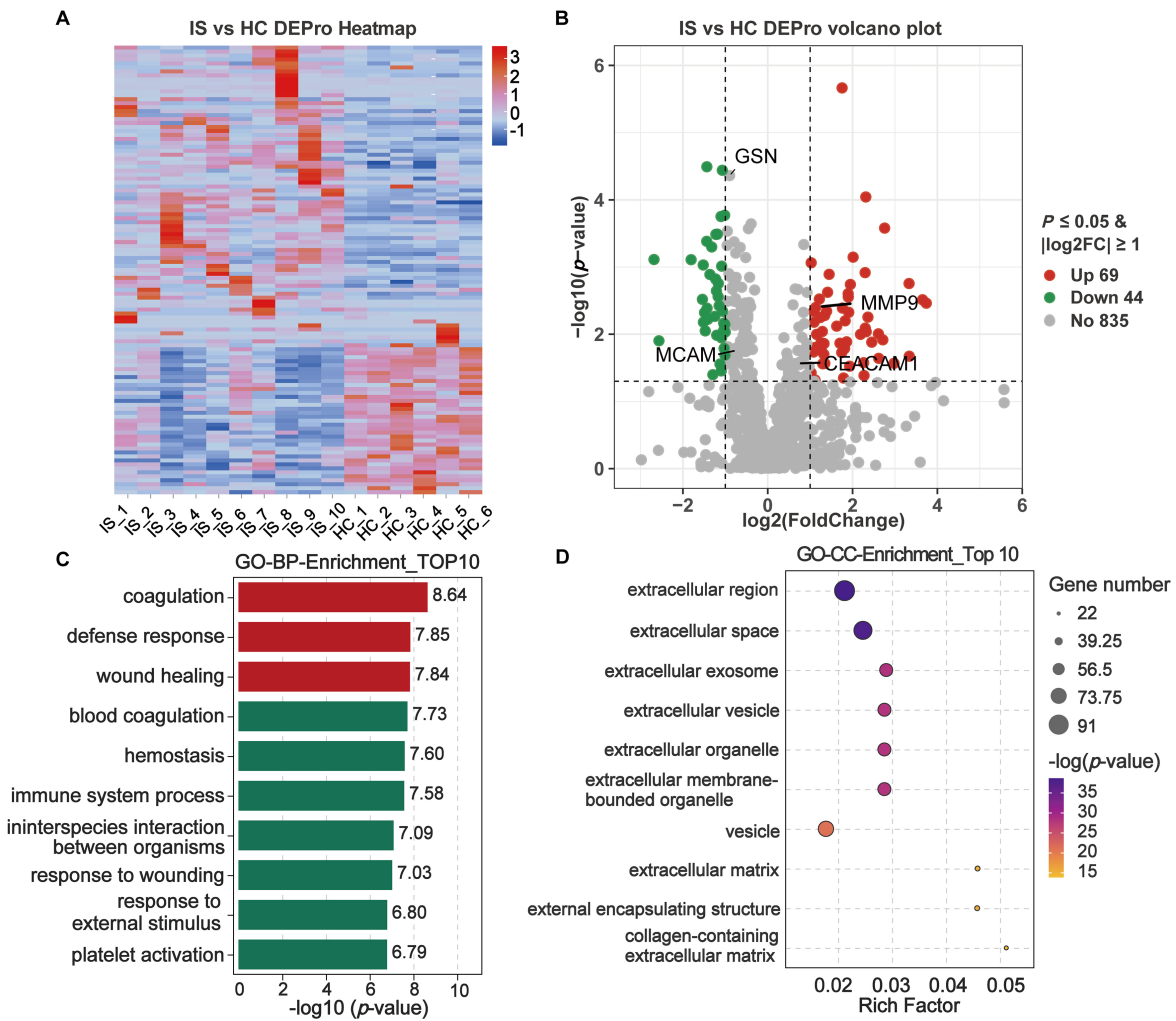
Pathologic alterations in IS-associated serum EVs in the discovery subset using proteomics. Human serum samples in the discovery subset were subjected to LC-MS/MS. DEPros were considered with *P*-value < 0.05 and |log2foldchange| ≥ 1.0 between the IS and HC groups. (A and B) Heatmap clustering and volcano plot of DEPros screened from MS proteomics; (C and D) GO term analysis. Enrichment in the biological process and cellular component categories, revealing the main pathological pathways of stroke rehabilitation. IS: Ischemic stroke; HC: healthy control; DEPros: differentially expressed proteins; GO: Gene Ontology; BP: biological process; CC: cellular component.

Label-free LC-MS/MS proteomics of serum EVs in the discovery subset (10 IS, 6 HCs) identified 113 DEPros, comprising 69 upregulated and 44 downregulated proteins, which exhibited clear clustering differences between IS and HC groups [Figure 2A and B and Supplementary Table 2]. GO enrichment analysis revealed that the top enriched biological processes included coagulation, defense responses, and wound healing during the convalescent phase of stroke [Figure 2C]. Among the top 10 enriched cellular components, significant alterations were observed in EVs and exosomes, as well as in extracellular matrix (ECM)-related compartments [Figure 2D]. These findings suggest that EVs are actively involved in stroke recovery and support the potential of EV-associated proteins as prognostic biomarkers for rehabilitation outcomes.

### Profiling single-EV surface proteins by PBA reveals disease-shifted EV subpopulations and candidate markers

**Figure 3:**
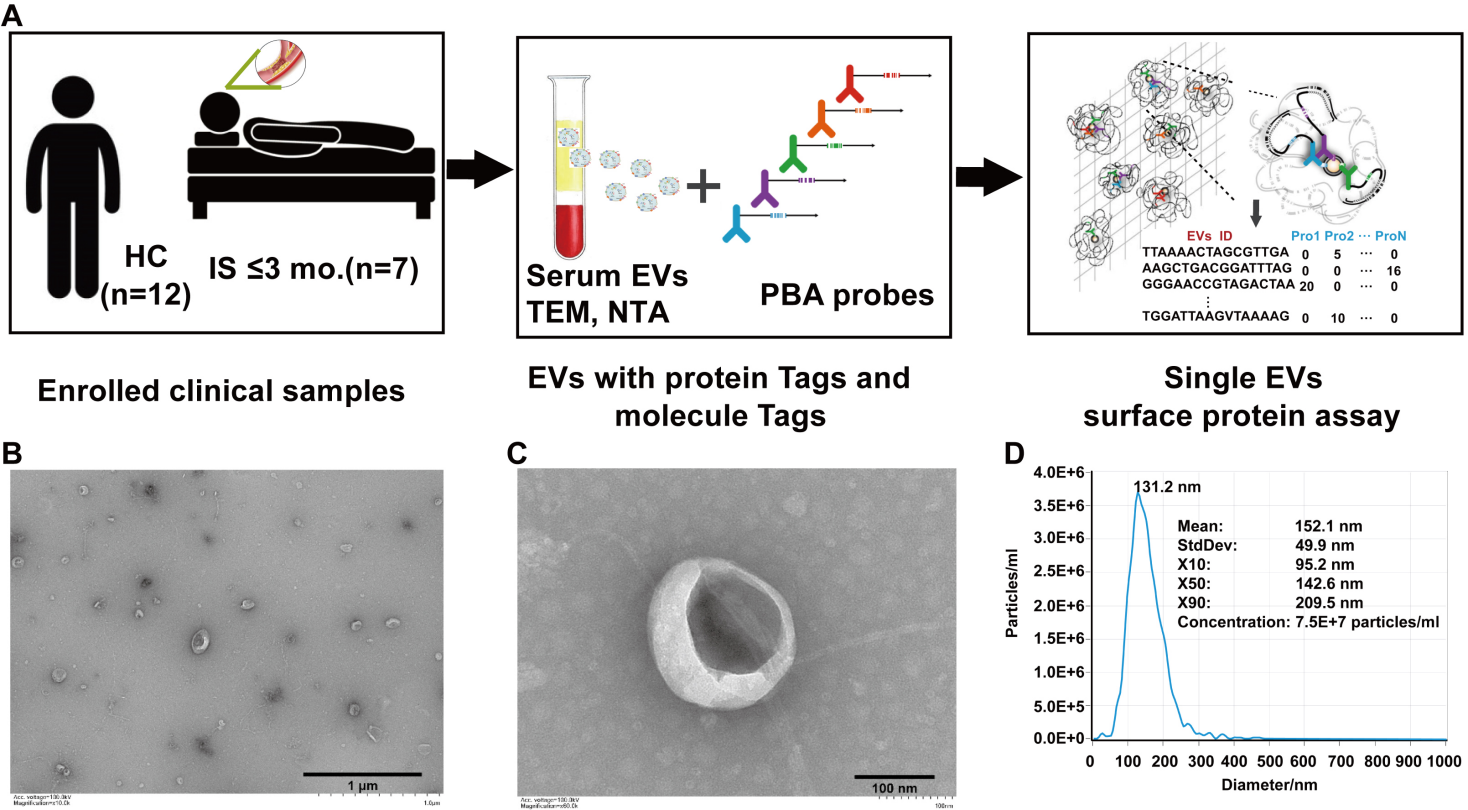
Profiling of IS-associated serum EVs surface proteins using PBA. (A) Flowchart of the study in the exploration subset. (B-C) Morphology of IS-associated serum EVs as recorded by TEM. Scale bars = 1 μm (B) and 100 nm (C). (D) NTA of serum-derived EVs from IS patients. The particle size distribution is presented as X10/X50/X90 (median diameter, X50). IS: Ischemic stroke; HCs: healthy controls; EVs: extracellular vesicles; TEM: transmission electron microscopy; NTA: nanoparticle tracking analysis; PBA: proximity barcoding assay.

To profile IS-associated EV surface protein heterogeneity at single-vesicle resolution, we applied PBA to the exploration subset (7 IS patients, 12 HCs), with comparable age distribution between groups [Table 1]. The PBA workflow is illustrated in Figure 3A. Serum EVs were enriched by ultracentrifugation and characterized by TEM and NTA, confirming the typical EV cup-shaped morphology and size distribution [Figure 3B-D]. In detail, NTA revealed the mean particle diameter was 152.1 nm with a standard deviation of 49.9 nm, and the particle concentration used for NTA measurement was 7.5 × 10^7^ particles/mL [Figure 3D].

**Table 1:** ADL assessment of enrolled subjects for PBA of EVs

**HCs** **(male; *n* = 12;** **average age = 56.25 ± 8.84 years)**			**Patients with IS** **(male; *n* = 7; average age = 58.14 ± 9.87 years)**				
Number	Sample name	Age	Patient number	Sample name	Age	Barthel index	Longshi Scale
HC_1_ID457	HCs101	45	IS_1_ID387	Sample38	58	45	Bedridden group
HC_2_ID458	HCs102	56	IS_2_ID402	Sample54	52	65	Bedridden group
HC_3_ID461	HCs105	39	IS_3_ID421	Sample73	51	45	Bedridden group
HC_4_ID462	HCs106	47	IS_4_ID426	Sample79	58	50	Bedridden group
HC_5_ID469	HCs109	63	IS_5_ID424	Sample77	74	35	Bedridden group
HC_6_ID471	HCs111	53	IS_6_ID394	Sample45	46	0	Bedridden group
HC_7_ID473	HCs113	59	IS_7_ID397	Sample49	68	10	Bedridden group
HC_8_ID474	HCs114	53					
HC_9_ID475	HCs115	68					
HC_10_ID476	HCs116	57					
HC_11_ID466	HCs119	65					
HC_12_ID467	HCs120	70					

ADL: Activities of daily living; PBA: proximity-dependent barcoding assay; EVs: extracellular vesicles; HCs: healthy controls; IS: ischemic stroke.

**Figure 4:**
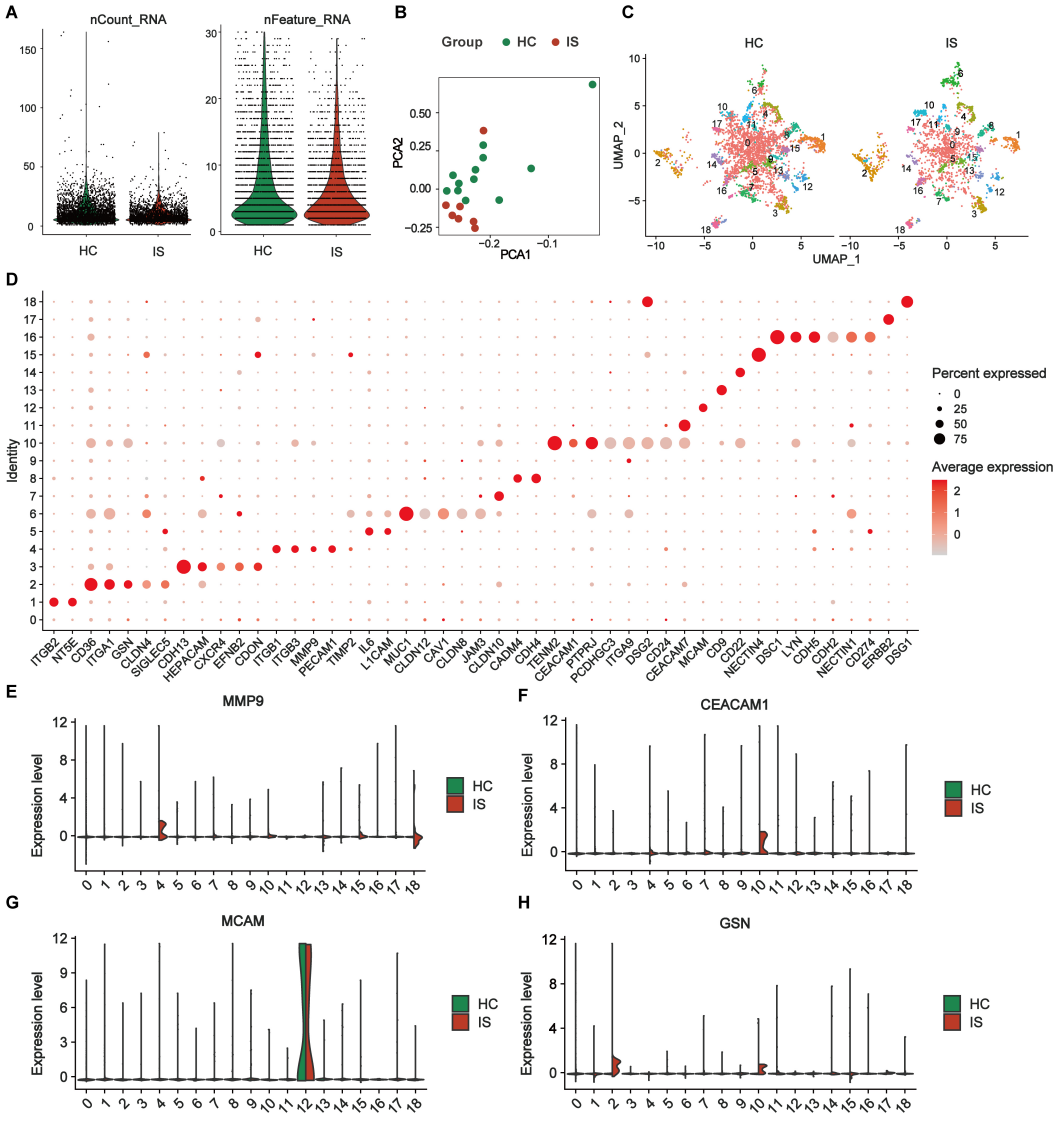
Differential expression profiles of single-EV surface proteins in the exploration subset. (A) Quality-control metrics of single-EV PBA data showing the distributions of nCount_RNA and nFeature_RNA after stringent EV-level filtering (Filter 2.2; EVs retained only if ≥ 2 proteins had detected expression ≥ 2) comparing HCs and IS; (B) PCA of SCTransform-normalized, Harmony-integrated EV surface protein profiles; (C) UMAP visualization of the integrated dataset shown separately for HCs and IS, revealing 19 EV subpopulations (Seurat clusters 0-18); (D) Dot plot of representative cluster-enriched marker proteins identified by Seurat FindMarkers (Wilcoxon rank-sum test; avg log2-fold change ≥ 0.25; Benjamini-Hochberg adjusted *P* < 0.05). For each cluster, up to six top-ranked markers are shown (all markers are displayed when a cluster contains ≤ 6 significant markers). Dot size indicates the percentage of EVs expressing the marker within each cluster; dot color indicates average normalized expression; (E-H) Ridge plots showing the distribution of normalized expression levels for key marker proteins (MMP9, CEACAM1, MCAM, and GSN) across EV clusters 0-18, split by group (HCs *vs*. IS). Exploration subset: IS (*n* = 7) and HCs (*n* = 12). IS: Ischemic stroke; HCs: healthy controls; EVs: extracellular vesicles; PBA: proximity barcoding assay; PCA: principal component analysis; MMP9: matrix metalloproteinase 9; CEACAM1: carcinoembryonic antigen-related cell adhesion molecule 1; MCAM: melanoma cell adhesion molecule; GSN: gelsolin.

Following stringent EV-level quality filtering (Filter 2.2), nCount_RNA (total detected counts per EV) and nFeature_RNA (number of detected features per EV) showed comparable distributions between IS and HCs [Figure 4A]. Principal component analysis (PCA) of the integrated surface-protein profiles suggested partial group-level separation [Figure 4B]. After SCTransform normalization and Harmony-based integration, UMAP resolved 19 EV subpopulations (clusters 0-18) [Figure 4C and Supplementary Figure 1A]. Cluster-enriched marker proteins were identified using Seurat Find Markers and are summarized in Supplementary Table 3 and representative markers visualized in Figure 4D, supporting that PBA profiling captured broad differential surface protein patterns between HC and IS EVs at the subpopulation level. Within these IS-enriched clusters, MMP9, CEACAM1, MCAM, and gelsolin (GSN) were among the enriched markers highlighted in the cluster-level summaries [Figure 4E-H and Supplementary Table 3]. Consistently, UMAP feature mapping showed that EVs with detectable expression of these proteins were predominantly localized to the corresponding cluster regions and exhibited group-skewed distributions when visualized separately for HCs and IS [Supplementary Figure 1B-F]. In addition, Western blotting of serum EV preparations confirmed detectable signals for MMP9, CEACAM1, MCAM, and GSN, together with canonical EV markers (CD63 and TSG101), while showing minimal signal for the endoplasmic reticulum marker (Calnexin) [Supplementary Figure 2]. These results motivated downstream targeted quantification to assess their clinical utility.

### MMP9 and CEACAM1 as promising biomarkers for predicting IS rehabilitation outcomes

**Figure 5:**
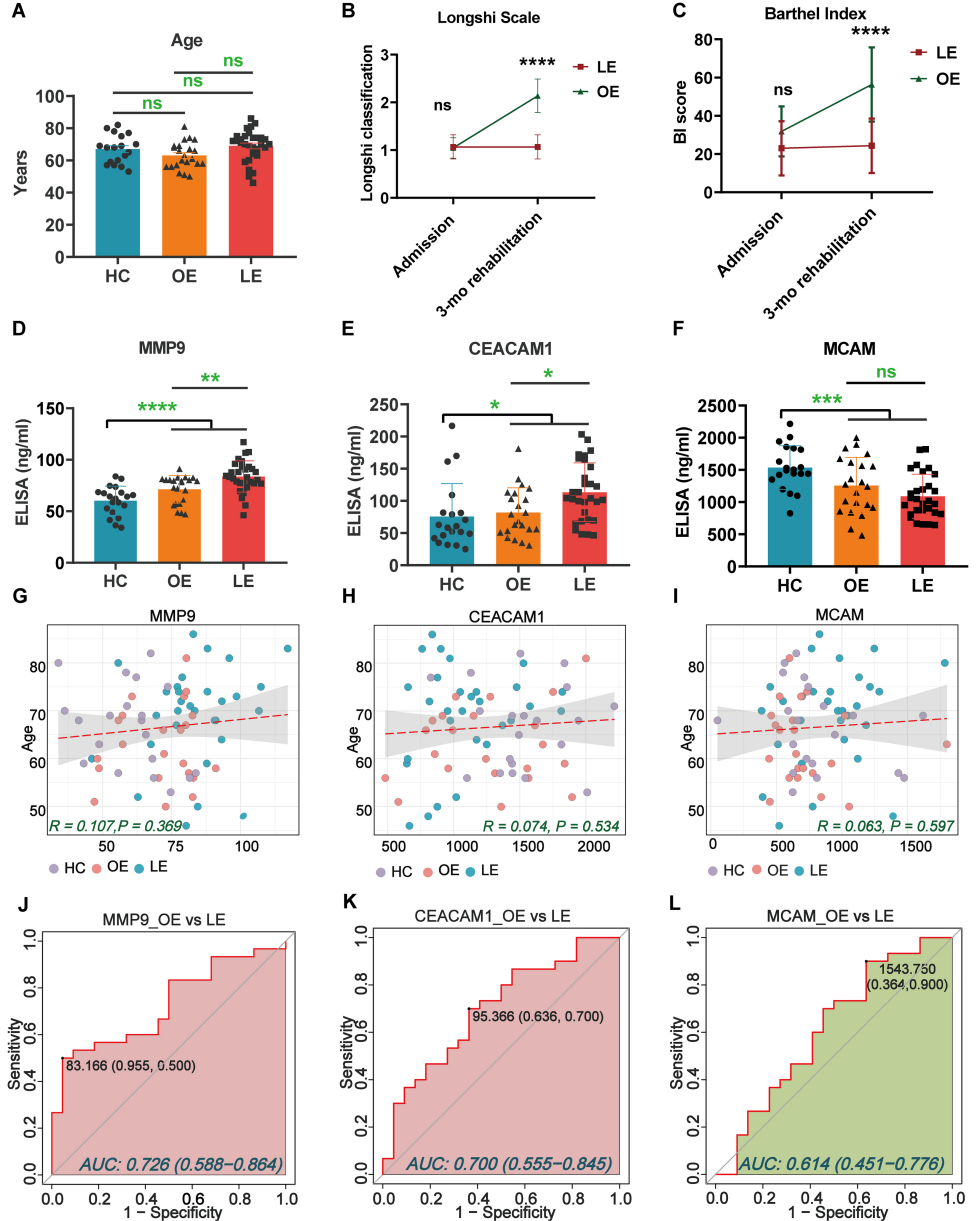
Biomarker identification using ELISA and ROC analysis in the validation cohort of patients with IS. The OE and LE classifications were based on 3-month Longshi Scale outcomes, where OE indicates patients who improved from Bedridden to Domestic or Community, and LE indicates those who remained Bedridden. (A) Distribution of age among the enrolled participants; (B and C) ADL assessment of the enrolled patients at admission and after 3 months by Longshi scale (1 = Bedridden group, 2 = Domestic group, 3 = Community group) and BI in the LE and OE groups. Each dot represents an individual; (D-F) Bar graphs showing serum protein levels of the three target molecules (MMP9, CEACAM1, and MCAM) among recruited participants in the LE (*n* = 30), OE (*n* = 22), and HCs (*n* = 20) groups. Data are presented as mean ± SD, with individual data points overlaid on the bars to illustrate the distribution of measurements; (G-I) Correlation scatter plots of target molecules *vs*. age. Each dot represents an individual. The data were analyzed using one-way ANOVA. ^*^P < 0.05; ^**^P < 0.01; ^***^P < 0.001; and ^****^P < 0.0001; (J-L) ROC analyses were performed as a binary classification using serum protein levels as continuous predictors. ROC curve analysis of MMP9, CEACAM1, and MCAM to distinguish between individuals in the LE and OE recovery groups. The black dots represent points on the ROC curve to gain optimal differentiation measures. BI: Barthel Index; LE: little-effect recovery group; OE: obvious-effect recovery group; HCs: healthy controls; IS: ischemic stroke; ROC: receiver operating characteristic curve; MMP9: matrix metalloproteinase 9; CEACAM1: carcinoembryonic antigen-related cell adhesion molecule 1; MCAM: melanoma cell adhesion molecule.

To validate the candidate biomarkers identified via proteomic and PBA analysis, serum samples from additional clinical cohort subgroups were analyzed using ELISA. The age distribution was comparable across three groups [Figure 5A and Table 2]. ADL recovery significantly differed between the LE and OE groups, as assessed by the Longshi Scale and BI [Figure 5B and C]. ELISA results confirmed that serum levels of MMP9 and CEACAM1 were significantly elevated in IS patients compared to HCs [Figure 5D and E and Supplementary Table 4], while MCAM levels were significantly reduced [Figure 5F]. Notably, MMP9 and CEACAM1 levels were further increased in the LE group compared to the OE group, indicating that elevated levels of MMP9 and CEACAM1 are negatively associated with functional recovery. In contrast, GSN levels did not show significant differences across groups [Supplementary Figure 3].

**Table 2:** ADL assessment of subjects included in the ELISA validation cohort

**Characteristic**	**HCs**	**Ischemic stroke**	
		**OE**	**LE**
Gender (male)	20	22	30
Age (year)	66.65 ± 8.83	63.05 ± 8.23	69.00 ± 9.60
Diagnosis	NA	Ischemic stroke	Ischemic stroke
BI_admission BI_3-mo rehabilitation	NA NA	31.82 ± 13.05 (56.36 ± 19.41)^**^	23.00 ± 14.18 (24.33 ± 14.25)
Longshi Scale_admission Longshi Scale_3-mo rehabilitation	NA NA	Bedridden group (Domestic group)	Bedridden group (Bedridden group)

^**^P < 0.01. ADL: Activities of daily living; ELISA: enzyme-linked immunosorbent assay; HCs: healthy controls; IS: ischemic stroke; OE: obvious-effect recovery group; LE: little-effect recovery group; BI: Barthel Index; NA: not applicable.NA indicates not applicable.

Linear regression analysis indicated no significant correlation between candidate biomarker levels and age (Pearson’s R < 0.2, *P* > 0.05; Figure 5G-I). ROC analysis showed that MMP9 (AUC = 0.726) and CEACAM1 (AUC = 0.700) had moderate sensitivity and specificity for distinguishing LE from OE patients [Figure 5J and K], whereas MCAM (AUC = 0.614) exhibited limited predictive value [Figure 5L]. Furthermore, in a multivariable binary logistic regression model adjusting for age and baseline disability (BI_admission), both MMP9 (OR = 1.067, 95%CI: 1.009-1.127, *P* = 0.023) and CEACAM1 (OR = 1.020, 95%CI: 1.002-1.037, *P* = 0.025) remained independently associated with functional outcome [Table 3]. Collectively, these results suggest that EV-associated MMP9 and CEACAM1 may serve as promising prognostic biomarkers for functional outcomes in patients undergoing post-ischemic stroke rehabilitation.

**Table 3:** Multivariable binary logistic regression analysis for predictors of functional outcome after ischemic stroke

**Variables**	**β (B)**	**S.E.**	**Wald χ^2^**	**OR**	**95%CI for OR**	***P* value**
Age	0.042	0.038	1.241	1.043	0.968-1.124	0.265
BI_admission	-0.065	0.029	5.162	0.937	0.886-0.991	0.023^*^
MMP9	0.065	0.028	5.203	1.067	1.009-1.127	0.023^*^
CEACAM1	0.020	0.009	4.993	1.020	1.002-1.037	0.025^*^

^*^P < 0.05. β (B) indicates the regression coefficient. The dependent variable was coded as LE = 1 (little-effect recovery group) and OE = 0 (obvious-effect recovery group). BI: Barthel Index; MMP9: matrix metalloproteinase 9; CEACAM1: carcinoembryonic antigen-related cell adhesion molecule 1; OR: odds ratio; CI: confidence interval; S.E.: standard error.

## DISCUSSION

The heterogeneity of EVs provides abundant and valuable insights into the biological complexity of stroke recovery, revealing disease-specific molecular alterations that bulk proteomics or transcriptomic analyses may obscure. By profiling surface proteins at the single-EV level and validating their expression in IS patients, we identified MMP9 and CEACAM1 as promising prognostic biomarkers for stroke rehabilitation. Our findings provide novel evidence supporting the utility of EV-derived surface proteins in predicting functional outcomes during the subacute phase of IS.

Exosomes can dynamically reflect disease onset and progression by carrying biologically active molecular cargo. Early diagnostic and prognostic approaches using EVs as biomarkers were primarily limited to bulk-level analyses, which attempted to associate the overall molecular content of EVs with pathological states. However, these approaches often yielded suboptimal results^[17]^. To better capture the heterogeneity of individual EVs, various advanced techniques have been developed and applied for single-EV analysis, including optical trapping^[18]^, Raman spectroscopy^[19,20]^, flow cytometry^[21]^, and cyclic imaging^[22]^. These studies consistently demonstrate the intrinsic heterogeneity of EVs and their capacity to carry disease-specific biomarkers, highlighting their significant potential in clinical diagnosis, prognosis, and therapeutic intervention. In this study, we utilized the recently developed PBA technique with antibody-DNA conjugates and next-generation sequencing to simultaneously profile approximately 184 types of adhesion molecules at the single-EV level in human serum. PBA has made notable progress in the exploration of aging and tumor-related disease biomarkers. For instance, single-EV analysis of noninvasive urine samples using PBA identified an Alzheimer’s disease-associated EV subpopulation, with signature proteins PLAU (urokinase-type plasminogen activator), ANXA1 (annexin A1), and ITGAX (integrin subunit alpha X), which could diagnose Alzheimer’s disease patients in blinded datasets with 88% accuracy^[23]^. In addition, PBA analysis of small EVs also revealed a TACSTD2-positive EVs subpopulation enriched in aging and tumor serum, which could serve as a dual biomarker for aging and tumors. Together, these advances establish a solid methodological foundation for interrogating disease-relevant EV subpopulations at single-vesicle resolution in clinical samples.

Several methodological considerations should be acknowledged when interpreting the present findings. Although EVs were isolated using differential ultracentrifugation without additional purification steps such as size-exclusion chromatography or density gradient centrifugation, the primary objective of this study was not to achieve maximal EV purity per se, but to interrogate EV-associated surface protein signals relevant to post-stroke recovery. To this end, we employed a single-EV resolved PBA, which profiles surface proteins based on vesicle-level co-localization rather than bulk protein abundance. This analytical framework substantially mitigates the confounding effects of soluble or co-isolated plasma proteins, as signal generation requires physical association of target proteins on the same EV membrane. Nevertheless, the absence of orthogonal purification strategies and complementary EV purity markers represents a limitation of the current study. Future investigations should integrate multi-step EV isolation approaches along with independent single-vesicle analytical platforms and functional assays to further strengthen EV specificity and to elucidate the mechanistic roles of EV-associated proteins in post-stroke repair and rehabilitation.

In the present study, we adopted a stepwise and hierarchical analytical strategy to bridge EV discovery with clinically feasible validation. First, unbiased serum proteomics was applied to identify EV-related pathways associated with stroke recovery. Second, single-EV surface protein profiling using PBA enabled the identification of disease-relevant EV subpopulations and candidate surface markers at vesicle resolution. Finally, due to current technical and sample availability constraints, targeted validation was performed using ELISA in an expanded clinical cohort, focusing on the overall serum abundance of selected candidates. This design reflects a pragmatic translational workflow rather than a strict one-to-one correspondence between vesicle-level measurements and bulk protein quantification. Within this analytical framework, proteomic analysis of serum from IS patients and HCs revealed that post-stroke pathological progression is closely associated with EV-related pathways. By integrating LC-MS/MS, single-EV profiling using PBA and ELISA validation, we demonstrated that serum levels of MMP9 and CEACAM1, identified as EV-associated candidates through single-EV analysis, were moderately predictive of rehabilitation outcomes, effectively distinguishing patients in the LE and OE recovery groups. These observations are consistent with previous studies that have identified MMP9 as a key biomarker for early diagnosis and prognostic evaluation of cerebrovascular diseases^[24,25]^. Elevated serum MMP9 levels have been shown to predict hemorrhagic transformation following IS^[26]^ and are associated with long-term risks of stroke recurrence and mortality over a 10-year follow-up period^[27]^. More recently, Kowalski *et al*. (2023) presented the first evidence of a rapid elevation of exosomal MMP-9 during the hyper-acute phase of stroke, providing compelling evidence for the temporal dynamics of post-stroke neuroinflammation. Their findings suggest that exosomal MMP9 may offer superior specificity and sensitivity for early diagnosis and risk stratification compared to its plasma-derived counterpart^[28]^. CEACAM1 has also emerged as a molecule of interest due to its anti-inflammatory role in vascular disease, primarily through inhibition of MMP9 activity^[29]^. Collectively, these findings derived from single-EV surface protein profiling using PBA support the potential of EV-associated MMP9 and CEACAM1 as prognostic biomarkers in stroke rehabilitation. Importantly, our study demonstrates that disease-relevant molecular signals can be traced to specific EV subpopulations.

To place these prognostic findings into a biological context, and within the scope of existing evidence, we next discuss the potential mechanistic roles of MMP9 in post-stroke injury and recovery. MMP9 is a key regulator of ECM remodeling and plays a dual role in the pathophysiology of IS by disrupting the BBB, initiating inflammatory cascades, and exacerbating neuronal injury. During the acute phase (within 7 days), MMP9 is rapidly upregulated and peaks within 24 h, leading to BBB disruption, cerebral edema, neuroinflammation, and neuronal apoptosis. In contrast, during the subacute phase (7-14 days), sustained MMP9 expression contributes to ECM remodeling and angiogenesis, facilitating tissue repair^[26]^. This dual role underscores the importance of temporal control in MMP9-mediated signaling. While several studies have shown that pharmacological inhibition of MMP9 mitigates early ischemic injury, improper timing may hinder reparative processes^[30]^. Furthermore, the interplay between MMP9 and its endogenous inhibitor TIMP1 (tissue inhibitor of metalloproteinases 1) is critical for maintaining ECM homeostasis. Dysregulation of the MMP9/TIMP1 balance has been linked to poor outcomes following stroke^[31,32]^, suggesting that therapeutic strategies aimed at restoring this balance may enhance rehabilitation effectiveness. Exploring therapeutic strategies to modulate MMP9 activity, either by direct inhibition or by restoring the MMP9/TIMP1 balance, may offer new avenues for improving stroke rehabilitation outcomes. Future studies should also investigate the mechanistic pathways through which MMP9 affects neurovascular recovery, aiming to harness its reparative potential while minimizing its detrimental effects.

CEACAM1, a multifunctional adhesion molecule, has been implicated in IS through its association with neutrophil activity, inflammatory mediators, and the preservation of BBB integrity. A recent review summarizes CEACAM1 as a context-dependent regulator of vascular homeostasis, endothelial barrier function, and immune signaling, with expression changes observed under hypoxia-related and aging-associated inflammatory conditions^[33]^. Reported effects of CEACAM1 on angiogenic and immunomodulatory pathways appear to be highly context specific and may depend on cellular source (e.g., endothelial cells *vs*. neutrophils/monocytes), isoform usage (long *vs*. short cytoplasmic tails, L/S), and the inflammatory milieu associated with aging [e.g., TNF-α (tumor necrosis factor alpha)-driven “inflammaging”]. Mechanistic links between CEACAM1 and pro-angiogenic signaling [including reciprocal interactions with VEGF-A/VEGFR2 (vascular endothelial growth factor A/vascular endothelial growth factor receptor 2)] have been described mainly in tumor biology and broader vascular/ischemic settings^[33]^, whereas stroke-specific evidence to date more consistently supports its role in inflammatory modulation and BBB homeostasis. Clinically, elevated CEACAM1 levels during different clinical stages of IS have been linked to increases in plasma inflammatory mediators, such as IL-10 (interleukin 10), MMP-9, and NGAL (neutrophil gelatinase-associated lipocalin)^[29]^. In a review by Sobey and Drummond (2013), CEACAM1 was highlighted as an adhesion molecule that restricts neutrophil transmigration across the BBB, thereby reducing inflammatory damage to brain tissue^[34]^. Notably, CEACAM1 is a pivotal factor in stroke-related BBB protection and inflammatory regulation, primarily through its modulation of the MMP-9/TIMP-1 axis. Preclinical studies in mouse models of IS have demonstrated that CEACAM1 exerts a protective effect by limiting neutrophil-mediated inflammatory responses, reducing MMP-9 secretion, and preventing the degradation of endothelial cells and basement membranes^[35,36]^. These effects collectively mitigate BBB damage following IS. Moreover, a study by Yu *et al*. (2020) revealed that CEACAM1 targets the MMP-9/TIMP-1 axis to inhibit the activation of the IκBα/NF-κB (inhibitor of nuclear factor kappa B alpha/nuclear factor kappa B) signaling pathway in diabetic atherosclerosis^[37]^. Future research should investigate the dynamic changes in CEACAM1 expression during stroke and their correlation with clinical outcomes, as well as the development of CEACAM1-targeted modulation of the MMP-9/TIMP-1 balance and suppression of inflammatory cascades. Collectively, these findings support CEACAM1 as a potential homeostatic regulator of post-stroke inflammation and BBB integrity, with possible links to neurovascular remodeling that warrant direct validation in stroke-specific models.

In clinical practice, improvements in ADL, such as changes in activity ability and activity range, are the most direct indicators of rehabilitation benefits. Currently, the BI and the Modified Rankin Scale are widely used ADL assessment tools in stroke recovery research and therapy^[38]^. In our study, we used the Longshi Scale to evaluate ADL improvements from admission to 3 months after rehabilitation. In addition, the Longshi Scale was approved by the National Standards Commission of China in 2016 as an evaluation method of self-care ability with respect to ADL among disabled people (GB/T37103-2018). The classification of patients into LE (Bedridden-to-Bedridden) and OE (Bedridden-to-Domestic/Community) groups was highly consistent with BI-based stratification. Notably, functional improvement from Bedridden to Domestic status requires integrated enhancement of multiple physiological systems. This was reflected in significant differences in neuroinflammatory, nutritional, metabolic, and neuromuscular markers, as well as in the levels and functions of several EV-associated proteins^[39,40]^. The newly recognized Longshi Scale, a visual-based, reliable, and easily implemented tool, may contribute to providing favorable evidence for marker research in stroke rehabilitation.

This study has several limitations. Sex differences in stroke pathophysiology and recovery have been well documented^[41,42]^, with women generally exhibiting greater post-stroke disability and distinct responses to vascular risk factors and therapeutic interventions. The restriction of our cohort to male patients minimized hormonal and physiological variability but inevitably limits the extrapolation of our findings to female populations. Future studies should therefore include both male and female participants to determine whether the predictive value of EV-associated biomarkers such as MMP9 and CEACAM1 for post-stroke recovery is consistent across sexes, and to explore potential sex-specific molecular signatures in EVs. The relatively small sample size, particularly in the discovery and exploration subsets, may also limit the robustness and generalizability of the findings; therefore, subsequent studies should perform formal sample size and power calculations based on the effect sizes observed here (e.g., AUC values of 0.726 and 0.700 for MMP9 and CEACAM1) to ensure adequate statistical power for validation. In addition, without size-exclusion chromatography or density-gradient purification, a small degree of non-vesicular protein carryover cannot be completely excluded; the EV-associated candidates identified using PBA have not been independently verified with alternative single-EV analytical methods, which would further strengthen reproducibility. Imaging-derived lesion metrics (e.g., infarct volume and location) were not available in the current dataset and therefore could not be incorporated into multivariable adjustment or incremental prediction analyses. Future prospective studies integrating standardized neuroimaging measures will be important to further confirm the independence and added predictive value of these EV biomarkers beyond established clinical and radiological predictors. Although EV-associated MMP9 and CEACAM1 were significantly associated with functional recovery, the underlying causal mechanisms remain unclear; larger clinical cohorts combined with mechanistic and experimental investigations are needed to validate these findings and elucidate the functional roles of EV-associated proteins in post-stroke repair and rehabilitation.

In conclusion, this study establishes MMP9 and CEACAM1 as EV-associated biomarkers for predicting rehabilitation outcomes in patients with IS. By integrating single-EV proteomic profiling with functional recovery evaluation, our findings lay the groundwork for more targeted, personalized rehabilitation strategies. However, they contribute to a better understanding of the molecular mechanisms underlying stroke recovery.

## DECLARATIONS

### Availability of data and materials

All relevant data are available from the authors upon reasonable request. Sequencing data in csv files are publicly available by https://ngdc.cncb.ac.cn/omix/select-edit/OMIX011139.

### Copyright

© The Author(s) 2026.
