# Supplementary material for: Verification of the predictive value of EV-associated biomarkers MMP9 and CEACAM1 in rehabilitation of ischemic stroke
Source: Extracell Vesicles Circ Nucl Acids. 2026 Apr 13;7(2):496–513. doi: 10.20517/evcna.2025.122 (PMC13174194; doi:10.20517/evcna.2025.122)
Supplement: Supplementary file 1 [file evcna-7-2-496-SupplementaryMaterials.zip › evcna60122-SupplementaryMaterials/evcna60122-SupplementaryMaterials.pdf]

Supplementary Materials

Verification of the predictive value of EV-associated biomarkers MMP9 and CEACAM1 in rehabilitation of ischemic stroke

Jiao Luo[1,2,#], You Cai[3,4,#], Yanling Cai[5,#], Chunxia Zhang[1], Ankang Liu[1], Yongyang Huo[1], Xuehui Fan[2], Ruixue Ye[2], Hong Gao[6], Meiling Huang[2], Xiaohua Zhang[1], Mingchao Zhou[2], Yulong Wang[1,2]

[1]Department of Rehabilitation Medicine, Dapeng New District Nan'ao People's Hospital, the First Affiliated Hospital of Shenzhen University, Shenzhen 518100, Guangdong, China.
[2]Department of Rehabilitation Medicine, the First Affiliated Hospital of Shenzhen University/the Second People's Hospital of Shenzhen, Shenzhen 518035, Guangdong, China.
[3]Department of Cell Biology and Neuroscience, Division of Life Sciences, School of Arts and Sciences, Rutgers, The State University of New Jersey, Piscataway, NJ 08854, USA.
[4]Shenzhen Institute of Translational Medicine, the First Affiliated Hospital of Shenzhen University/the Second People's Hospital of Shenzhen, Shenzhen 518038, Guangdong, China.
[5]Shenzhen Institute of Translational Medicine, the First Affiliated Hospital of Shenzhen University, Shenzhen Second People's Hospital, Shenzhen SecreTech Co., Ltd., Shenzhen 518129, Guangdong, China.
[6]Department of Pharmacy, the Eighth Affiliated Hospital of Sun Yat-sen University, Shenzhen 518000, Guangdong, China.
[#]These authors contributed equally to this work.

Correspondence to: Prof. Yulong Wang, Dr. Mingchao Zhou, Department of Rehabilitation Medicine, the First Affiliated Hospital of Shenzhen University/the Second People's Hospital of Shenzhen, Shenzhen 518035, Guangdong, China. E-mail: wangyulong@szu.edu.cn; zhoumc06@email.szu.edu.cn

Supplementary Methods

Western blots validation of serum EV purity and EV-associated MMP9/CEACAM1

Serum extracellular vesicles (EVs) were isolated from independent samples of healthy controls (HC), ischemic stroke patients with little recovery (LE), and those with obvious recovery (OE). For this validation, 10 subjects per group were included; 2 mL serum was collected per subject from remaining clinical laboratory serum. To increase EV yield and minimize individual variability, sera were pooled with 5 subjects per pool, generating two pooled EV samples per group.

EVs were isolated by differential ultracentrifugation. Briefly, serum was cleared of debris and large particles by centrifugation, followed by ultracentrifugation at 100,000 × g to pellet EVs. The EV pellet was washed in PBS and re-pelleted by ultracentrifugation, then resuspended in lysis buffer for protein extraction. Total protein concentration was determined using a BCA assay. EV lysates were normalized and prepared at 1.5 μg/μL, and 25 μg total protein per lane was loaded for SDS-PAGE and Western blotting. Electrophoresis was performed at 40 V for 30 min followed by 120 V for 70 min. Proteins were transferred to membranes at 260 mA for 100 min. Membranes were blocked and incubated with primary antibodies against EV markers, negative markers, and candidate proteins as follows: CEACAM1 (Affinity; DF6564; ~120 kDa), MMP9 (Proteintech; 10375-2-AP; ~92 kDa), MCAM (Abcam; ab75769; ~113 kDa), Calnexin (Proteintech; 10427-2-AP; ~90 kDa), GSN (Affinity; DF7117; ~86 kDa) CD63 (Abcam; ab217345; 25-65 kDa), and TSG101 (Abcam; ab125011; ~45 kDa). After incubation with HRP-conjugated secondary antibodies, immunoreactive bands were visualized using chemiluminescence.

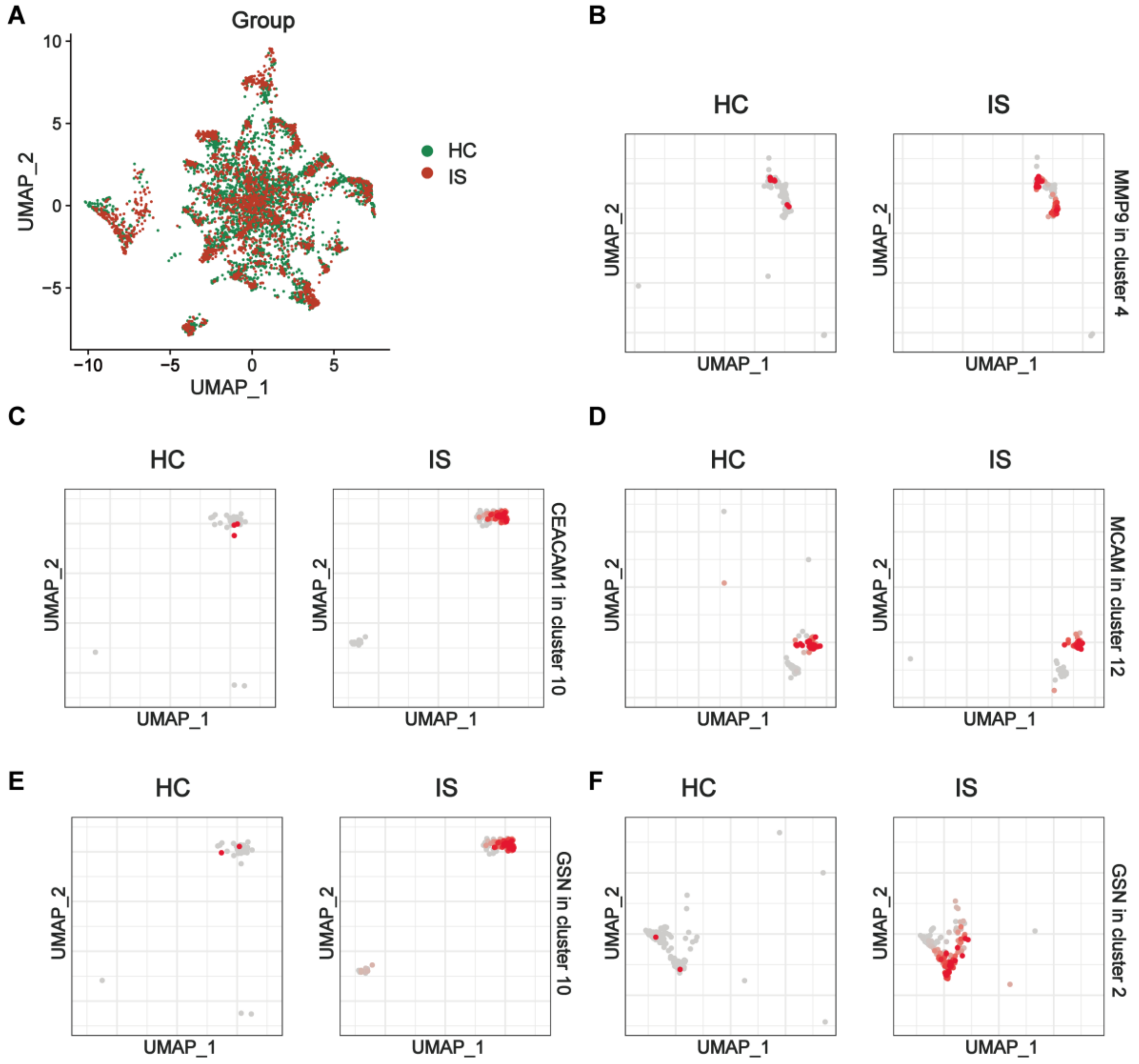

Supplementary Figure 1. Group distribution and feature mapping of key marker proteins across the integrated UMAP embedding corresponding to Figure 4. (A) UMAP of Harmony-integrated EVs colored by group (HC vs IS); (B-F) UMAP feature plots displayed separately for HC and IS, highlighting EVs with detected expression (red) over all EVs (grey) for selected markers associated with disease-shifted subpopulations: MMP9 (cluster 4), CEACAM1 (cluster 10), MCAM (cluster 12), and GSN (clusters 2 and 10). All plots are based on EVs passing Filter 2.2 and SCTransform normalization with Harmony integration. Exploration subset: IS (n=7) and HC (n=12). HC: Healthy control; IS: ischemic stroke; CEACAM1: carcinoembryonic antigen-related cell adhesion molecule 1; MMP9: matrix metallopeptidase 9; MCAM: melanoma cell adhesion molecule; GSN: Gelsolin.

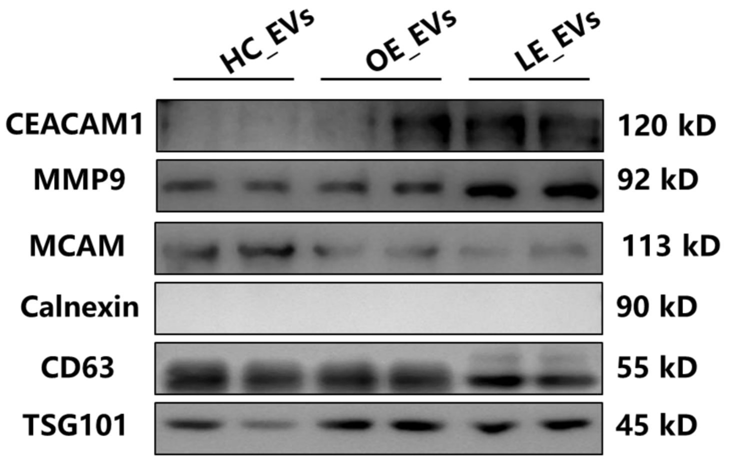

Supplementary Figure 2. Western blots characterization of serum EV preparations and EV-associated candidate proteins. EV lysates from pooled serum samples (HC, LE, OE; two pools per group, 5 subjects per pool) were probed for EV markers (CD63, TSG101), a negative marker (Calnexin), and candidate proteins (MMP9, CEACAM1, MCAM, and GSN). CD63 and TSG101 were detected in EV preparations, while CALNEXIN in was not detected, supporting EV enrichment and limited ER contamination. MMP9 and CEACAM1 were detectable in serum EV lysates, supporting their association with EV preparations. LE: Little-effective recovery group; OE: obvious-effective recovery group; HC: healthy control; CEACAM1: carcinoembryonic antigen-related cell adhesion molecule 1; MMP9: matrix metallopeptidase 9; MCAM: melanoma cell adhesion molecule; GSN: Gelsolin; TSG101: Tumor susceptibility gene 101 protein.

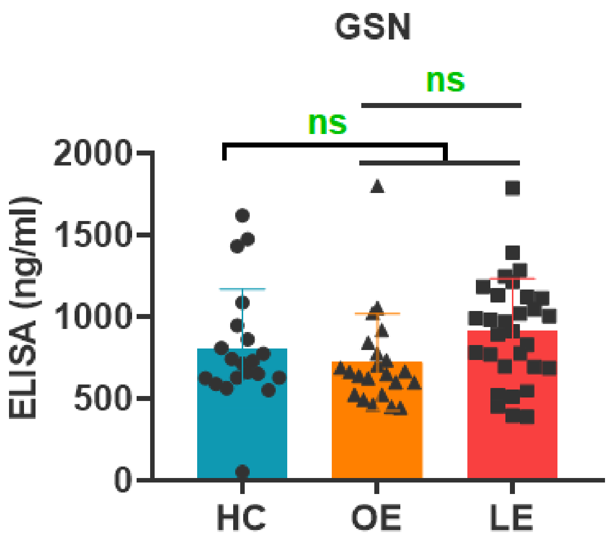

Supplementary Figure 3. Supplementary figure corresponding to Figure 5. Histograms of serum protein levels for the three target molecules among recruited participants in the LE (n=30), OE (n=22), and HC (n=20) groups. Each point represents an individual. Data are presented as mean ± SD. LE: Little-effective recovery group; OE: obvious-effective recovery group; HC: healthy control; GSN: Gelsolin.
